# Local hero: A phase II study of local therapy only (stereotactic radiosurgery and / or surgery) for treatment of up to five brain metastases from HER2+ breast cancer. (TROG study 16.02)

**DOI:** 10.1016/j.breast.2024.103675

**Published:** 2024-02-05

**Authors:** Claire Phillips, Mark B. Pinkham, Alisha Moore, Joseph Sia, Rosalind L. Jeffree, Mustafa Khasraw, Anthony Kam, Mathias Bressel, Annette Haworth

**Affiliations:** a Peter MacCallum Cancer Centre, Melbourne, Australia; b Sir Peter MacCallum Department of Oncology University of Melbourne, Parkville, Australia; c Department of Radiation Oncology, Princess Alexandra Hospital, Brisbane, Australia; d Faculty of Medicine, University of Queensland, Brisbane, Australia; e Trans-Tasman Radiation Oncology Group, Newcastle, Australia; f Department of Neurosurgery, Royal Brisbane and Women's Hospital, Brisbane, Australia; g Royal Melbourne Hospital, Parkville, Australia; h The Alfred, Prahran, Australia; i Monash University, Clayton, Australia; j Department of Physics, University of Sydney, Sydney, Australia

**Keywords:** HER2+ breast cancer, Brain metastases, Radiosurgery, Whole brain radiotherapy, HER2-Targeted therapy

## Abstract

Introduction, A decade ago, stereotactic radiosurgery (SRS) without whole brain radiotherapy (WBRT) was emerging as preferred treatment for oligometastatic brain metastases. Studies of cavity SRS after neurosurgery were underway. Data specific to metastatic HER2 breast cancer (MHBC), describing intracranial, systemic and survival outcomes without WBRT, were lacking. A Phase II study was designed to address this gap.

Method, Adults with MHBC, performance status 0–2, ≤ five BrM, receiving/planned to receive HER2-targeted therapy were eligible. Exclusions included leptomeningeal disease and prior WBRT. Neurosurgery allowed ≤6 weeks before registration and required for BrM >4 cm. Primary endpoint was 12-month requirement for WBRT. Secondary endpoints; freedom from (FF-) local failure (LF), distant brain failure (DBF), extracranial disease failure (ECDF), overall survival (OS), cause of death, mini-mental state examination (MMSE), adverse events (AE).

Results, Twenty-five patients accrued Decembers 2016–2020. The study closed early after slow accrual. Thirty-seven BrM and four cavities received SRS. Four cavities and five BrM were observed. At 12 months: one patient required WBRT (FF-WBRT 95 %, 95 % CI 72–99), FFLF 91 % (95 % CI 69–98), FFDBF 57 % (95 % CI 34–74), FFECDF 64 % (95 % CI 45–84), OS 96 % (95 % CI 74–99). Two grade 3 AE occurred. MMSE was abnormal for 3/24 patients at baseline and 1/17 at 12 months.

Conclusion, At 12 months, SRS and/or neurosurgery provided good control with low toxicity. WBRT was not required in 95 % of cases. This small study supports the practice change from WBRT to local therapies for MHBC BrM.

## Highlights

• HER2+ breast cancer oligo-brain metastases in patients receiving HER2-targeted therapy.
• HER2+ brain metastases are well controlled by stereotactic radiosurgery and or neurosurgery.
• 12 months after radiosurgery and or neurosurgery, most patients are alive and functioning well.
• Whole brain radiotherapy is rarely required up to 12 months after radiosurgery and or neurosurgery.

## 1 Introduction

Brain metastases (BrM) are common in metastatic HER2-positive breast cancer (MHBC) [1]. Whilst newer agents such as tucatinib and trastuzumab deruxtucan show promising intracranial activity, surgery and or radiation therapy still play an important role in treatment of BrM [2,3]. MHBC patients with good performance status can have prolonged survival after diagnosis of BrM so durable intracranial disease control and minimisation of late treatment toxicity are important management goals [4].

This study was conceived in 2014, when only trastuzumab and lapatinib were available for MHBC in Australia. Symptomatic BrM were a common problem in the clinic and development of BrM despite good extracranial disease (ECD) control was a well-recognised challenge [5]. Despite this, the predicted median survival of good performance status patients with BrM was 15–24 months [5,6]. Based on data from mixed-histology studies, stereotactic radiosurgery (SRS) without whole-brain RT (WBRT) was accepted for treatment of up to four BrM [7,8]. There was limited prospective data on outcomes for patients with MHBC BrM specifically [6]. In particular, data correlating intracranial outcomes (including risk of leptomeningeal disease) with: systemic therapy use, status of ECD and cause of death were lacking. Thus, MHBC-specific information was needed to better inform patients of expected treatment outcomes and also to underpin future therapeutic study design. This phase II clinical trial was designed to evaluate the need for WBRT after local therapy (neurosurgery and/or SRS) only in a cohort of MHBC patients with long predicted median survival.

## 2 Method

**Fig. 1:**
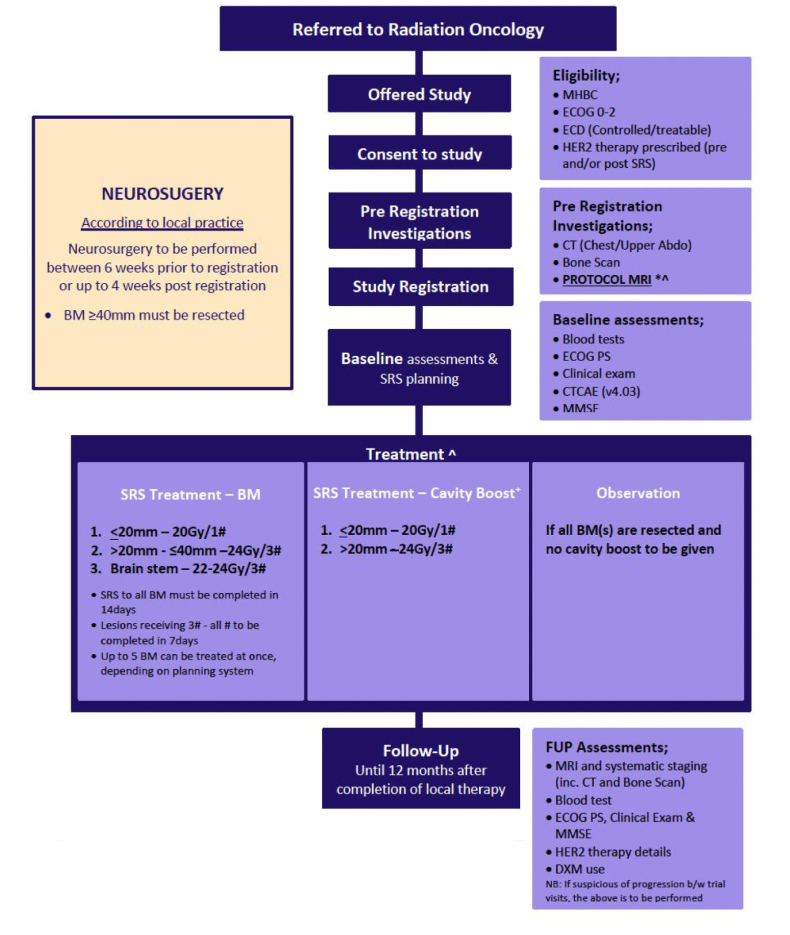
Trial schema.

This multicentre prospective phase II clinical trial was conducted by the Trans-Tasman Radiation Oncology Group (TROG) across ten participating sites. Hospital Research Ethics Committee approval was obtained and all participants gave written informed consent. The study was registered with Clinical Trials.gov (NCT02898727). The trial schema in Fig. 1 summarises timelines from participant registration to imaging and then treatment.

### 2.1 Eligibility

Eligible participants were aged ≥18 years with Eastern Cooperative Group (ECOG) PS 0–2 and up to five untreated untreated BrM from MHBC. At least one lesion needed to be suitable for surgery and/or SRS. If not already receiving HER2-targeted therapy, this was to commence within four weeks of local BrM treatment. BrM size and volume criteria were as follows: maximal dimension of an unresected metastasis (es) < 40 mm, maximum volume of any single in situ BrM planning target volume (PTV) ≤10 cm^3^, any single surgical cavity PTV ≤15 cm^3^ cavity and summated PTV of all lesions to be treated with SRS (cavity and in situ) ≤15 cm^3^ [9]. Exclusion criteria were pregnancy, diffuse leptomeningeal disease and prior WBRT.

### 2.2 Treatment

Neurosurgery was performed according to usual practice at each centre and permitted up to six weeks prior to study registration. SRS was delivered according to a radiotherapy quality assurance protocol developed for TROG studies. Linac, Cyberknife and Gamma Knife platforms were allowed. Centre credentialling (end-to-end test) and planning of a benchmarking case was required. All SRS plans were reviewed retrospectively for compliance (authors MP and CP). Broadly, for in situ tumours the gross tumour volume (GTV) was visible tumour on MRI and planning CT, using the larger if the volume was larger on CT. The margin from GTV to planning target volume (PTV) was 1 mm for Linac and Cyberknife and 0 mm for Gamma Knife. For cavities, the clinical target volume (CTV) was the contrast-enhanced resection cavity excluding the surgical track and any contrast-enhanced areas suspicious for residual disease. The CTV to PTV margin was 2 mm for Linac/Cyberknife and 0 mm for Gamma Knife. The SRS dose was 20 Gy in a single fraction ( ± 2 Gy) for lesions <20 mm and 24 Gy in 3 fractions for cavities or lesions ≥20 mm. 99 % of the PTV was to receive the treatment dose. Comprehensive radiotherapy treatment planning data was collected in DICOM format (Digital Imaging and Communications in Medicine) for all patients however analysis of this technical data is beyond the scope of this clinical manuscript. BrM <8 mm and surgical cavities without residual disease could be observed at the discretion of the treating clinician. Three fraction treatments were to be delivered within 7 days. All SRS was to be completed within 14 days of SRS commencement. A guideline for when to consider WBRT or other local therapy in the management of intracranial progression after initial therapy was included in the protocol but not mandated (see Appendix A. We note that practice has changed since this guideline was written.).

### 2.3 Assessments

Baseline assessments included history and examination, volumetric contrast enhanced MRI brain, computerised tomography chest/upper abdomen, bone scan, the Mini-mental state examination (MMSE) [10] and systemic therapy details. A preferred MRI brain protocol was developed but not mandated (Appendix B). Other imaging was according to local imaging protocols. The MMSE was chosen as a simple cognitive function screen and a score of ≥26 was chosen as normal. All assessments were repeated at three, six, nine and twelve months after local therapy. A protocol amendment allowed the bone scan not to be repeated every three months where prior scans had been stable and there was no clinical or CT suggestion of progressive extracranial disease.

### 2.4 Endpoints

The primary endpoint was the requirement for WBRT at 12 months. Secondary endpoints were distant brain failure, local brain failure, ECD failure, pattern of first failure, overall survival, cause of death, Common Terminology Criteria for Adverse Events version 4.0 (CTCAEv4) [11] and MMSE score. Serious Adverse Events were reported according to the International Conference on Harmonisation Guideline for Good Clinical Practice [12].

A modified Response Assessment in NeuroOncology (RANO) BrM approach was used for intracranial disease assessments in that lesions were assessed individually, whereas RANO-BrM combines all lesions in a single disease status [13]. As per RANO, lesions ≥10 mm were measurable, lesions <10 mm unmeasurable and if radiation necrosis was suspected, clinical correlation and early follow-up imaging (4–6 weeks) was to be undertaken with the final diagnosis (local brain failure or radionecrosis) to be back-dated once confirmed.

### 2.5 Statistics

A pragmatic sample size of 50 patients to be accrued over two years. This sample size was chosen based on the prevalence of the study population at the time and in consideration of the calculated 95 % confidence intervals for different WBRT rates at 12 months, including allowance for up to 4 % of cases lost to follow-up. For a 20 % rate of WBRT, the 95 % CI would be 10–35 % and for 30 % WBRT it would be 17–44 %. The Kaplan-Meier method was used to estimate the time to event endpoints at the key time points and an event chart (swimmers plot) was provided to visually describe when the events occurred. The maximum grade of each adverse event for each participant was described in tabular form as counts and percentages. MMSE scores were tabulated at each visit. All statistical analysis were performed in R version 4.2.1 [14].

## 3 Results

Between December 2016 and December 2020, 25 patients were accrued. The study was closed before reaching the accrual target of 50 because of slow accrual. One patient was excluded after registration but before SRS because the cavity PTV was >15 cm^3^ leaving 24 patient datasets for analysis. The median age was 57.6 years (range 30.4–75.1) and median number of BrM was one (range 1–5). All patients had previously received targeted HER2-therapy, either in the adjuvant setting and or for metastatic disease. Baseline patient characteristics are shown in Table 1.

**Table 1:** Baseline Characteristics.

	Total (n = 24)
Age, years	
Mean (SD)	57.3 (13.2)
Median [range]	57.6 [30.4–75.1]
IQR	48.0–69.3
**ECOG, n (%)**	
0	9 (38 %)
1	10 (42 %)
2	5 (21 %)
**Time from initial diagnosis of BC to registration, years**	
Mean (SD)	3.4 (2.5)
Median [range]	3.2 [0.1–10.2]
IQR	1.5–4.8
**Prior systemic therapy, n (%)**	
HER2-targeted therapy	24 (100 %)
Chemotherapy	22 (92 %)
Hormone therapy	9 (38 %)
**Number of brain lesions, n (%)**	
1	11 (46 %)
2	5 (21 %)
3	5 (21 %)
4	1 (4 %)
5	2 (8 %)
**Extra cranial disease (ECD) status, n (%)**	
Absent	7 (29 %)
Controlled on HER2-targeted therapy	11 (46 %)
Treatable with HER2-targeted therapy	6 (25 %)
**Extracranial disease sites**	
Bone	13 (54 %)
Liver	11 (46 %)
Lung	8 (33 %)
Lymph Node	6 (25 %)
Other	4 (17 %)

### 3.1 Local treatment

There were 42 in situ BrM and eight surgical cavities in 24 patients. Thirty-seven in situ BrM were treated with SRS whilst five unmeasurable lesions (all five mm or less) were observed. Four surgical cavities were treated with SRS and four were observed (Table 2). At the time of analysis, it was noted that one patient had undergone neurosurgery and cavity SRS (30 Gy in 5 fractions) at another centre prior to registration. The patient received SRS to one in situ BrM after registration per protocol. As the surgery had been within six weeks of rereferral to a study centre, both lesions were included in the analysis and the cavity SRS dose noted as a protocol violation. All other SRS plans were reviewed and no major protocol variations were found. The PTV was not available for the protocol-violation cavity nor for the observed in situ BMs for which no SRS plans were generated.

**Table 2:** Local treatment.

Treatment, n (%)	n = 50
In situ SRS	37 (74 %)
In situ Observed	5 (10 %)
Neurosurgery alone	4 (8 %)
Cavity SRS	4 (8 %)
**SRS PTVs, cc**	**n = 41**
Mean (SD)	2.1 (3.3)
Median [range]	0.6 [0.0–14.4]
IQR	0.3–2.2
Missing	1
**SRS dose, n (%)**	**n = 41**
18Gy in 1 fraction	3 (7 %)
20Gy in 1 fraction	24 (59 %)
22Gy in 1 fraction	4 (10 %)
24Gy in 3 fractions	9 (22 %)
30Gy in 5 fractions	1 (2 %)

### 3.2 Endpoints

**Fig. 2:**
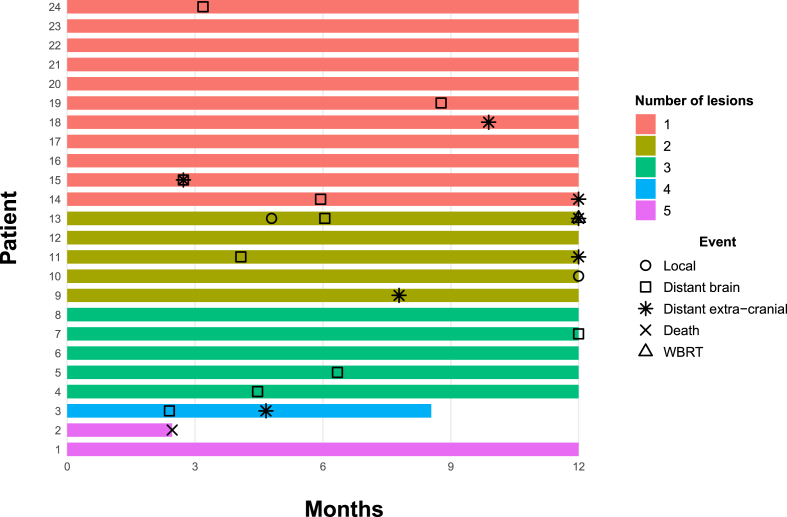
Event Chart (Swimmers plot).

**Fig. 3:**
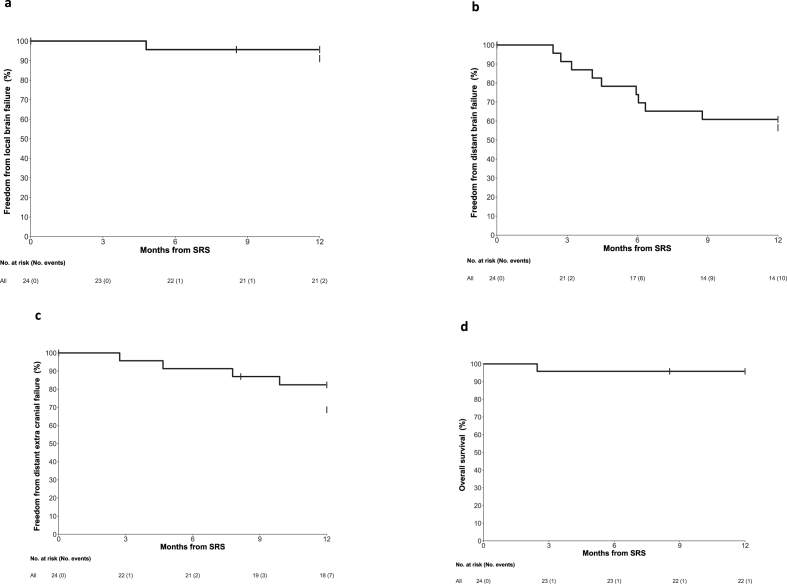
Kaplan Meier plots for time-to-event endpoints, 3a Freedom from local failure, 3b Freedom from distant brain failure, 3c Freedom from extracranial disease failure, 3d Overall Survival.

Fig. 2 displays the number of treated lesions for each participant and the sequence of relevant events (WBRT, local brain failure, distant brain failure, extracranial disease failure and death). Table 3 and Fig. 3 present Kaplan-Meier estimates and plots for each time-to-event endpoint. There is no Kaplan-Meier plot for WBRT as there was only one event. The observed rate of WBRT was well below the possible rates that were predicted at the time of study design. Two patients did not complete 12 months follow-up; one patient died from extracranial disease progression before the 3-month assessment and a second withdrew to receive best supportive care for progressive extracranial disease. One patient required WBRT during the follow-up period (Freedom from WBRT 95 %, 95 % CI 72–99). The patient developed local recurrence in an irradiated right cerebellar cavity 4.5 months after cavity SRS and was initially observed on trastuzumab-emtansine. Leptomeningeal disease developed in the posterior fossa at six months and was treated with posterior fossa radiotherapy but the disease continued to progress and ultimately WBRT was required for extensive leptomeningeal disease 12 months after registration. A second event meeting RANO criteria for local brain failure occurred 12 months after SRS. Longer follow-up will be needed to determine if this was true failure or radionecrosis. One of the five observed in situ lesions progressed at three months and was treated with SRS. Distant brain failure occurred in ten patients, five of which also had extracranial disease failure with or after the distant brain failure. An extra two patients had extracranial failure without intracranial progression.

**Table 3:** Time To Event Endpoints.

Months after local therapy	Freedom from WBRT	Freedom from local failure	Freedom from distant brain failure	Freedom from distant extra-cranial failure	Overall Survival^a^
3	100	100	91 (69, 98)	96 (73, 99)	96 (74, 99)
6	100	96 (73, 99)	74 (51, 87)	91 (69, 98)	96 (74, 99)
9	100	96 (73, 99)	61 (38, 77)	87 (65, 96)	96 (74, 99)
12	95 (72, 99)	91 (69, 98)	57 (34, 74)	69 (45, 84)	96 (74, 99)

a A second patient entered best supportive care at 8 months. Date of death unknown.

### 3.3 HER2-targeted treatment

HER2-targeted therapy received during the study period is shown in Table 4. Twenty-one patients were already receiving targeted therapy prior to registration and three started within four weeks of registration. Two of the 21 had a change of HER2-therapy from registration to three months, without documented disease progression other than the diagnosis of BrM at registration. One case with both extracranial and distant brain failure at three months had a change of HER2-targeted therapy at 12 months. Another case with distant brain failure at three months had a systemic therapy change at 12 months. The case that required WBRT had commenced trastuzumab emtansine within four weeks of registration and was changed to lapatinib when intracranial disease progressed despite posterior fossa radiotherapy. Three of the seven extracranial disease failure events occurred at 12 months, potentially triggering a change to HER2-targeted therapy after the 12- month visit.

**Table 4:** HER2-targeted therapy.

	Total (n = 24)
HER2 therapy at trial start	
Pertuzumab + Trastuzumab	19 (79 %)
Trastuzumab	1 (4 %)
Trastuzumab deruxtecan	1 (4 %)
Trastuzumab Emtansine	3 (12 %)
**Change of HER2 therapy**	
No	19 (79 %)
Yes	5 (21 %)
**What was the change in HER2 therapy**	
Pertuzumab + Trastuzumab to Trastuzumab emtansine prior to 3 month visit	2 (40 %)
Trastuzumab emtansine to Lapatinib prior to 9 month visit Pertuzumab + Trastuzumab to Trastuzumab emtansine prior to 12 month visit	1 (20 %) 1 (20 %)
Trastuzumab emtansine to Trastuzumab prior to 12 month visit	1 (20 %)

### 3.4 Adverse events

Twenty patients experienced at least one adverse event attributable to local treatment and for which the worst grade was CTCAE Grade 1 in 14 (58 %), Grade 2 in 4 (17 %) and Grade 3 in two (8 %) patients respectively (Table 5). The grade 3 events were a post-operative seizure and an unusual allergic skin reaction. The latter was attributed to the antiseptic wash and/or local anaesthetic used for fixation of the SRS frame and warranted admission for overnight observation. During the study period there was one clinical case of CTCAE grade 2 radionecrosis (Recorded as headaches in Table 5.). Three months after SRS, the patient developed recurrent headaches. MRI findings were equivocal for progression on RANO criteria. The symptoms resolved after 4 weeks of corticosteroids and imaging improved, in keeping with a clinical diagnosis of RN (grade 2). The patient was also receiving a PD-L1 inhibitor on a clinical trial.

**Table 5:** Adverse Events attributable to local treatment

Grade (n = 24)				
Adverse Events	1	2	3	Total
Allergic Skin Reaction	0	0	1	1 (4 %)
Seizure	0	0	1	1 (4 %)
Headache	9	2	0	11 (46 %)
Fatigue	14	2	0	16 (67 %)
Memory Impairment	2	1	0	3 (12 %)
Other	11	0	0	
Any grade 3 or worse			2	2 (8 %)

### 3.5 Mini-mental state examination

MMSE scores are tabulated in Table 6. Completion rates are noted for each timepoint. The majority of patients retained a normal score over time. MMSE was below normal for three of 24 patients at baseline, two of which had normal scores after baseline. There were no assessments after baseline for the third of these patients. One of 17 patients had an abnormal score at 12 months. This patient had a normal score at baseline and had developed both extracranial and distant brain failure during follow-up.

**Table 6:** Mini-mental state examination.

Score	Baseline (n = 24)	FUP 3 m (n = 20)	FUP 6 m (n = 17)	FUP 9 m (n = 16)	FUP 12 m (n = 17)
21	1 (4 %)	0	0	0	0
22	0	0	0	1 (6 %)	0
23	2 (8 %)	0	0	0	0
25	0	1 (5 %)	0	0	1 (6 %)
26	4 (17 %)	1 (5 %)	1 (6 %)	2 (12 %)	0
27	3 (12 %)	0	1 (6 %)	1 (6 %)	0
28	0	3 (15 %)	1 (6 %)	2 (12 %)	2 (12 %)
29	5 (21 %)	4 (20 %)	6 (35 %)	0	6 (35 %)
30	9 (38 %)	11 (55 %)	8 (47 %)	10 (62 %)	8 (47 %)

## 4 Discussion

There are several reasons to avoid WBRT for oligometastatic BrM where possible. These are; reduced acute and late toxicity by not exposing the whole brain to RT, improved local control from the high radiation doses used in SRS, and protection of normal brain tolerance to radiation in case WBRT should be required at a later date for disease that is not amenable to local therapy, notably leptomeningeal disease. In the last 5 years, clinical practice has changed rapidly such that the use of local therapies, including cavity SRS, without WBRT has become common, at least for up to 10 BrM [9,15,16]. Nonetheless, this study, albeit small, is informative as one of the few prospective studies in a homogenous cohort of MHBC, with detailed reporting of targeted therapy use, extracranial disease status, and BrM outcomes.

The primary endpoint of the study was the need for WBRT during 12 months follow-up. Only one patient required WBRT, giving a freedom from WBRT of 95 % (95 % CI 45–84). Freedom from local brain failure was 91 % (95 % CI 69–98). Given the long expected median survival of this MHBC cohort, durable local control like this is an important endpoint for quality of life and potentially for overall survival [17,18]. In contrast, studies of WBRT demonstrate relatively poor local control in MHBC patients with BrM, even within the first 12 weeks after treatment [19]. WBRT can be repeated for intracranial progression but there is little data describing its tolerability beyond a median survival of around 6 months [20]. Taken together, these factors imply that premature use of WBRT should be avoided as it may not achieve durable local control of existing BrM and it may also compromise the ability to properly treat new intracranial disease that might occur at a later date.

As is expected with omission of WBRT [8,21] distant brain failure was the commonest disease recurrence event occurring in nine patients (57 %, 95 % CI 34–74). In addition, one in-situ lesion that had been observed at baseline grew sufficiently to be treated with SRS. WBRT was not required to manage any of this disease. The low rate of any additional SRS or neurosurgery therapy throughout 12 months of follow-up demonstrates that it is not always necessary to treat every MRI-apparent MHBC BrM when it is first detected. In this study, small BrM size was the only criterion used to allow observation rather than SRS. Whilst BrM size (volume) is a useful guide, the decision to observe is influenced by other factors including extent of associated oedema, eloquence of BrM location, tempo of BrM growth and intracranial activity of systemic therapies. The results of this study also support the underlying hypothesis of this study; that omission of upfront WBRT is preferred in most patients with oligometastatic BrM receiving HER2-targeted therapy. In the current era, particularly with the advent of centrally active HER2-targeted agents, the philosophy is taken even further in that it is preferable to omit WBRT unless symptomatic BrM cannot be treated safely without it [22].

Toxicity from local therapy was low in this small study. There were only two (8 %) grade 3 events and one case of RN (grade 2). Radionecrosis is the main long-term toxicity of SRS and is especially relevant for patients with long median survival such as those with MHBC. In a large US series (all histologies), the median time to radionecrosis was 7.6 months. Others report that radionecrosis can probably occur any time after SRS and is commonest at 12–18 months after SRS [23]. The extent to which HER2-targeted therapies increase the risk of radionecrosis is unclear [24,25] but a causal link was not apparent in this study.

The MMSE scores in Table 6 reflect the high functional status of many patients with brain oligometastases from MHBC. As this was a single arm study, there was no plan to rigorously assess neurocognition but the results are in keeping with prior studies which report better cognitive function after SRS compared with WBRT. Cognitive function is protected by use of SRS over WBRT, particularly in older patients [17,21]. A potential exception to this is a permanent deficit from radionecrosis affecting memory or cognitive pathways.

During the conduct of the study, access to HER2-targeted agents (outside of a clinical trial) changed considerably, with the addition of pertuzumab and trastuzumab emtansine to trastuzumab and lapatinib. The observed delay between documented progression and a change to HER2-targeted therapy likely reflects drug access rather than clinical need. This small study was unable to address a major question of interest at the time of study design, which was to analyse for any relationship between the status of extracranial disease and the development of new BrM. It can be noted that whilst 50 % of patients with distant brain failure did also develop extracranial disease failure, in all but one of these cases, the extracranial disease failure occurred months after the distant brain failure.

A major weakness of this study is its small size. It closed early after poor accrual. We postulate several reasons for this. Firstly, pertuzumab and trastuzumab-emtansine became available in Australia for MHBC. Treatment with these agents may have improved overall disease control and thus reduced the prevalence of MHBC BrM cases. Secondly, competing studies opened which enabled access to even newer HER2-targeted agents, in particular HER2 Climb [2]. Finally, it may be that the study did not offer sufficient interest to local investigators and their patients as practice was already shifting away from WBRT towards local therapies. The Covid pandemic played a minor role during 2020 when accrual to clinical trials was suspended at some centres. More particularly, the pandemic did affect the ability for accrued patients to complete MMSEs because follow-up appointments shifted to telehealth. Another limitation is the relatively short follow-up over 12 months. With more than 90 % patients alive at 12 months, longer follow-up would provide more meaningful information for all endpoints. Finally, this is a single arm study. At the time of study design there was no appetite to conduct a study of local therapy with or without WBRT. A prior randomised study of prophylactic cranial radiotherapy for MHBC had failed to accrue [26].

## 5 Conclusion

Local therapy with SRS and/or NS without WBRT provided good intracranial control with low toxicity in patients with MHBC and BrM. At 12 months 95 % of cases did not require WBRT. There was one documented death from ECD and none from intracranial disease. Although this study had a small sample size with relatively wide confidence intervals, the results support the clinical practice change away from WBRT to local therapies for oligometastatic BrM.

## Funding

The study received funding from 10.13039/100015277Roche Products Pty Ltd (for capitation) and from the Peter MacCallum Cancer Centre Foundation. The study was an investigator-initiated study with no input from Roche or any other company with regard to design, sponsorship, conduct or analysis. The study was fully sponsored by the Trans-Tasman Radiation Oncology Group TROG.

## Ethical approval

Hospital Research Ethics Committee approval was obtained and all participants gave written informed consent. The study was conducted according to the principles of Good Clinical Practice Guidelines.

## **ABC 7**th **international consensus conference 2023**

Best Poster Presentation, Patient Advocacy.

## CRediT authorship contribution statement

**Claire Phillips:** Writing – review & editing, Writing – original draft, Project administration, Methodology, Investigation, Funding acquisition, Conceptualization. **Mark B. Pinkham:** Writing – review & editing, Writing – original draft, Investigation. **Alisha Moore:** Writing – review & editing, Writing – original draft, Project administration, Methodology. **Joseph Sia:** Writing – review & editing, Writing – original draft, Investigation. **Rosalind L. Jeffree:** Writing – review & editing, Writing – original draft, Investigation, Conceptualization. **Mustafa Khasraw:** Writing – original draft, Methodology, Conceptualization. **Anthony Kam:** Writing – original draft, Conceptualization. **Mathias Bressel:** Writing – review & editing, Writing – original draft, Formal analysis, Conceptualization. **Annette Haworth:** Writing – original draft, Methodology, Conceptualization.

## Declaration of competing interest

No conflicting relationship exists for any author.

## Abbreviations ^1^

^1^ Brain metastases: (BrM)
Metastatic HER2-positive breast cancer: (MHBC)
Freedom from local failure: (FFLF)
Freedom from distant brain failure: (FFDBF)
Freedom from extracranial disease failure (FF ECDF)Mini-mental stat examination: (MMSE)
