# Supplementary material for: Local hero: A phase II study of local therapy only (stereotactic radiosurgery and / or surgery) for treatment of up to five brain metastases from HER2+ breast cancer. (TROG study 16.02)
Source: Breast. 2024 Feb 5;74:103675. doi: 10.1016/j.breast.2024.103675 (PMC10869940; doi:10.1016/j.breast.2024.103675)
Supplement: Multimedia component 1 [file mmc1.docx]

#### Appendix B – Local HERO study. Recommended MRI Protocol

A standardised MRI acquisition protocol is required to allow the use of imaging response as a surrogate for targeted treatment efficacy in brain metastases. A variety of imaging capabilities is expected in this multicenter trial (e.g. Field strength, gradient system, manufacturer, sequences). The aim of the MRI scanning protocol is to minimise image variability and optimise trial data reliability by using a vendor neutral standardize imaging protocol.

Initial assessment and subsequent follow-up MRI for each patient should be done using a scanner with the same field strength (all 1.5T or all 3T MRI), preferably by the same physical scanner using identical imaging parameters.

The standardised protocol is a balance between maximizing recruitment, compliance and data quality. Lesion contrast is highly dependent on sequence parameters. Image standardisation reduces measurement variability due to protocol differences. Minor hardware differences and sequence timing can result in significant change in image contrast. Timing between pre-contrast T1 weighted acquisition, intravenous gadolinium injection and post-contrast T1 weighted acquisition is critical. Gadolinium contrast dose also affects contrast enhancement.

The key difference between the standardised protocol and many existing clinical protocol is the use of pre and post contrast 3D T1 weighted imaging, the timing between the gadolinium contrast injection and the post contrast 3D T1 weighted acquisition.

Recommended MR Imaging Protocol for 1.5T and 3T scanners

- 1. 3D IR GRE T1 weighted Pre contrast
  2. Axial 2D T2 FLAIR
  3. Axial 2D DWI
  4. Gadolinium contrast injection
  5. Axial 2D T2 Weighted
  6. 3D IR GRE T1 weighted Post contrast
  7. SWI or T2* is desirable but not compulsory

Precontrast 3D isotropic IR-prepped T1 weighted gradient echo sequence (3D IR GRE), slice thickness less than or equal to 1.5mm, no interslice gap.

A 3D acquisition without inversion preparation will result in different contrast compared to MPRAGE or other IR-prepped 3D T1 weighted sequences and should be avoided.

3D IR GRE T1 = Inversion recovery gradient recalled echo sequence is equivalent to the following:

- Magnetization prepared rapid gradient echo (MPRAGE, Siemens & Hitachi)
- Inversion recovery spoiled gradient echo IR-SPGR or FSPGR with inversion activated (BRAVO, GE)
- 3D Turbo field echo (TFE, Phillips)
- 3D fast field echo (3D Fast FE, Toshiba)

Axial 2D or 3D T2 weighted FLAIR (fluid attenuated inversion recovery) sequence obtained using TSE readout, less than or equal to 5mm thick, no interslice gap.

Axial 2D DWI, 3 directional diffusion weighted imaging obtained using eco planar acquisition, less than or equal to 5mm thick, no interslice gap.

Intravenous gadolinium contrast injection, 0.1mmol/kg injection with a gadolinium chelated contrast agent. The use of a power injector is not compulsory but is desirable at an injection rate of 3-5 mls/second.

Axial 2D T2 weighted TSE sequence, less than or equal to 5mm thick, no interslice gap. TSE turbo spin echo (Siemens & Philips) is equivalent to FSE Fast spine echo (GE, Hitachi and Toshiba).

Post contrast 3D isotropic T1 weighted IR prepped gradient echo sequence should have equivalent parameters to the pre-contrast 3D T1 weighted images.

Advanced sequences (E.g. DTI, perfusion, spectroscopy) can be acquired after contrast injection as per local preference, clinical need and protocol, so long as the standardised 3D post contrast T1 weighted images are collected between 4-8 minutes after IV contrast gadolinium injection.

Susceptibility weighted imaging or T2* weighted Gradient echo recall sequence using the usual local protocol is desirable but not compulsory for the trial.
