# Supplementary material for: Local hero: A phase II study of local therapy only (stereotactic radiosurgery and / or surgery) for treatment of up to five brain metastases from HER2+ breast cancer. (TROG study 16.02)
Source: Breast. 2024 Feb 5;74:103675. doi: 10.1016/j.breast.2024.103675 (PMC10869940; doi:10.1016/j.breast.2024.103675)
Supplement: Multimedia component 2 [file mmc2.docx]

## APPENDIX A : Guidelines for use of WBRT at time of progression in the brain

The use of WBRT may not be necessary at time of disease progression in the brain. The decision to use further local therapy or WBRT is dependent on a number of variables including; ECOG performance status (PS), symptoms from the brain disease, number of brain lesions, time of brain failure, feasibility of further SRS or surgery at sites of local failure, status of extracranial disease (ECD) and availability of further HER2 targeted therapies.

Multidisciplinary meeting discussion of each case at the time of disease failure is strongly recommended.

Surgery is recommended for progressive for large and /or symptomatic lesions regardless of lesion number, as per normal clinical practice.

The following guideline should be adhered to as much as possible but is not binding. In cases where the guidelines cannot be followed, the reason for giving or not giving WBRT must be documented.

*If ECD has progressed but PS is good and another line of HER2 therapy can commence up to a maximum of 4 weeks after demonstration of progressive disease, then the participant is to be managed as for controlled ECD.*

#### ≤ 6 months (i.e. up to and including the 6 month follow-up MRI)

Progressive ECD with no targeted therapy options or PS > 2

*Any brain failure*

Brain is symptomatic WBRT / Consider best supportive care if poor PS

Brain is asymptomatic WBRT / Observation at discretion of treating team

Controlled / treatable ECD and PS 0-2

*Distant Brain Failure*

Brain symptomatic > 3 new lesions WBRT

≤ 3 new lesions LT should be considered first.

- If local therapy not technically feasible, WBRT

Brain asymptomatic As for symptomatic lesions or observation

If observation - repeat MRI at 6 weeks is recommended

(Observation may be considered if, for example, the new lesions are very small)

*Local failure only*

Brain symptomatic Further LT should be considered first

- eg resection if prior SRS, SRS if cavity failure has had no prior cavity SRS, repeat SRS

If LT not technically feasible - WBRT

Brain Asymptomatic As for symptomatic lesions or observation

If observation – a repeat MRI at 6-8 weeks is recommended

1. **> 6 months (i.e. after the 6 month follow-up MRI)** Progressive ECD with no targeted therapy options or PS > 2 *Any brain failure*

Symptomatic WBRT / consider best supportive care if poor PS Asymptomatic WBRT / Observation and MRI at discretion of treating team

Controlled /treatable ECD and PS 0-2

*Distant Brain Failure*

| Symptomatic | > 5 new lesions | WBRT |
| --- | --- | --- |
|  | ≤ 5 new lesions | LT should be considered first.   - If LT not technically feasible, consider change of |
|  |  | targeted HER2 therapy   - WBRT |

Asymptomatic As for symptomatic lesions or observation

If observation – a repeat MRI at 8-12 weeks is recommended (Observation may be considered if, for example, the new lesions are very small)

*Local failure only*

Symptomatic Further LT should be considered first

- eg resection if prior SRS, SRS if cavity failure has had no prior cavity SRS, repeat SRS

If LT not technical feasible - WBRT

Asymptomatic As for symptomatic lesions or observation

If observation - Repeat MRI at 8-12 weeks is recommended
